# MicroCT for comparative morphology: simple staining methods allow high-contrast 3D imaging of diverse non-mineralized animal tissues

**DOI:** 10.1186/1472-6793-9-11

**Published:** 2009-06-22

**Authors:** Brian D Metscher

**Affiliations:** 1 Department of Theoretical Biology, Gerd Müller, head, University of Vienna, Althanstraße 14, 1090 Austria

## Abstract

**Background:**

Comparative, functional, and developmental studies of animal morphology require accurate visualization of three-dimensional structures, but few widely applicable methods exist for non-destructive whole-volume imaging of animal tissues. Quantitative studies in particular require accurately aligned and calibrated volume images of animal structures. X-ray microtomography (microCT) has the potential to produce quantitative 3D images of small biological samples, but its widespread use for non-mineralized tissues has been limited by the low x-ray contrast of soft tissues. Although osmium staining and a few other techniques have been used for contrast enhancement, generally useful methods for microCT imaging for comparative morphology are still lacking.

**Results:**

Several very simple and versatile staining methods are presented for microCT imaging of animal soft tissues, along with advice on tissue fixation and sample preparation. The stains, based on inorganic iodine and phosphotungstic acid, are easier to handle and much less toxic than osmium, and they produce high-contrast x-ray images of a wide variety of soft tissues. The breadth of possible applications is illustrated with a few microCT images of model and non-model animals, including volume and section images of vertebrates, embryos, insects, and other invertebrates. Each image dataset contains x-ray absorbance values for every point in the imaged volume, and objects as small as individual muscle fibers and single blood cells can be resolved in their original locations and orientations within the sample.

**Conclusion:**

With very simple contrast staining, microCT imaging can produce quantitative, high-resolution, high-contrast volume images of animal soft tissues, without destroying the specimens and with possibilities of combining with other preparation and imaging methods. Such images are expected to be useful in comparative, developmental, functional, and quantitative studies of morphology.

## Background

As genomic, functional, and developmental evolutionary studies continue to expand far beyond the few traditional model species, the need for general, direct methods of accurate three-dimensional imaging of animal specimens has never been greater. Any comparative, functional, or ontogenetic analysis of morphology requires calibrated three-dimensional representation of anatomical structures with their natural shapes, orientations, and spatial relationships in as close to their natural state as specimen preparation can allow.

Methods for constructing 3D visualizations of animal specimens fall into two broad categories: those based on reconstruction from serial section images and those based on whole-volume imaging. The former set of methods require sectioning the specimen under study and then aligning images of those sections into a 3D dataset – a laborious process even in its recent computer-assisted and semi-automated forms [1]. Whole-volume imaging methods are generally non-destructive but have tended to be more specialized in their applications. MicroMRI has been used for some time to generate volumetric images of soft tissue morphology (e.g [2]), but it requires rather costly imaging equipment. Optical projection tomography (OPT) has been developed recently as a method for anatomical and molecular imaging [3]. Based on transmission of visible light, OPT requires more-or-less transparent samples, and it is finding a useful range of applications, such as localization and measurement of structures within a whole organ [4].

The oldest tomographic imaging method is x-ray computed tomography (CT) [5,6], now in widespread use for clinical imaging. X-ray microtomography (micro-computed tomography, or microCT) is identical in its basic principles to medical CT scanning and has been increasingly utilized in non-clinical research in the last eight years or so. A sample to be imaged is placed in the path of an x-ray beam so that it forms a projection image on a scintillator or other x-ray-sensitive detector array. The sample is rotated and imaged at a large number of angles, and the sequence of projection images is "back-projected" to reconstruct the x-ray absorption at each point within the scanned volume (see [5] and [6] for thorough treatments).

A volume image obtained from microCT (or any tomographic method) consists of a stack of reconstructed cross sections normal to the axis of rotation. Reconstruction programs usually generate voxels (volume pixels) that are isotropic, and the voxel dimensions are automatically calculated with the same accuracy as the imaging system's calibration. The numerical value computed for each voxel is a linear x-ray attenuation coefficient at the corresponding point in the sample volume [6]. Thus, a tomographic volume image is represented as a three-dimensional matrix of brightness values, equal to a stack of aligned two-dimensional digital images. Such datasets are becoming more and more useful and versatile with the growing sophistication and availability of 3D image viewing, manipulation, and analysis software.

MicroCT imaging systems can be divided into two general classes based on their x-ray sources: lab-based scanners and synchrotron systems. Lab-based scanners contain their own commercial or custom x-ray source, and a number of different systems are commercially available from several companies. The image resolutions achievable with these systems extend into the range of light microscopy, down to one or a few microns. Most of the self-contained scanning systems currently cost from around 120,000 to over 400,000 Euro.

Synchrotron-based microCT systems are capable of much finer resolutions because the synchrotron's electron stream can be used to generate high-brilliance x-ray beams that have narrow bandwidths at chosen energies and that can be manipulated with diffractive zone plates, analogous to focusing a light beam with refractive lenses in an optical microscope. Despite the obvious drawback that the system must be connected to a beamline at one of a few synchrotron facilities, synchrotron-based x-ray tomographic microscope systems have been used for non-destructive imaging of microfossils [7] and for sub-cellular imaging at resolutions of 60 nm and below [8-10]. The narrow-band beam is also more conducive to phase-contrast imaging than a broadband x-ray tube, affording one kind of soft-tissue contrast [11,12], but the contrasts produced by phase effects are much more pronounced in samples with steep phase gradients (i.e. sharper edges) rather than the softer gradients prevalent in most tissues.

MicroCT is already an established technology for imaging diverse mineralized animal tissues (reviewed in [13]), but the widespread application of microCT imaging in comparative morphology has been limited by the low intrinsic x-ray contrast of non-mineralized tissues. Although x-ray contrast enhancement agents are used routinely in clinical radiography, only a few techniques have appeared for imaging soft tissues in preserved animal specimens: a clinical radiographic contrast preparation containing organically-bound iodine has been shown to impart differential x-ray contrast to post-mortem mouse and rabbit brains [14]; osmium staining has been demonstrated successfully several microCT applications, including phenotyping mouse embryos with microCT [15] and imaging honeybee brains [16]; a reduced-silver nerve staining method has been used to image *Drosophila *brains with microCT [17]; and a contrast resin perfusion method has been used successfully by Wirkner et al. [18,19] for 3D imaging of arthropod circulatory systems.

With sufficient contrast imparted to soft tissues, linear and volumetric size changes in development can be measured readily, and comparisons of those measurements can be made between species or between control and genetically or experimentally manipulated specimens. Thus microCT offers a source of quantitative data for modeling of developmental and evolutionary changes, and microCT imaging is already being applied to quantitative studies of variation [20] and of development [21].

A previous report from this lab [22] highlights the utility of microCT for imaging vertebrate embryos, and the present report demonstrates the broad applicability of a few very simple and effective contrast stains for imaging various animal tissues using x-ray microtomography. These readily available, low-toxicity contrast agents open a wide range of microCT applications in comparative, developmental, and functional morphological research.

## Methods

### MicroCT imaging systems

Two commercial lab-based systems were used in this work. Smaller samples were imaged at higher resolutions using an Xradia MicroXCT system . This system uses a 90 keV/8 W tungsten x-ray source, a cooled 1 k × 1 k CCD camera, and switchable scintillator-objective lens units, which give fields of view from 5 mm down to a few hundred microns, with corresponding pixel sizes from 5 μm to less than 500 nm. The practical limit of resolution for images from this scanner is about 1 μm.

Samples larger than about 5 mm were imaged at lower resolutions with a SkyScan 1174 scanner  employing a 50 keV/40 W tungsten x-ray source and a 1.3 megapixel CCD camera. This scanner also has variable optical magnification of the scintillator panel, giving fields of view from 6 mm to about 30 mm, with voxel sizes from 6 μm to about 30 μm and actual spatial resolution limits from about 15 μm to 75 μm. This is the lowest-priced commercial microCT scanner (ca. 60,000 Euro) known to the author at the time of this writing.

All images were reconstructed using the software provided with the respective microCT systems. Images made on the Xradia MicroXCT were reconstructed with 2 × 2 pixel binning to improve signal-to-noise and reduce file sizes. This system's resident control, reconstruction, and viewing software uses its own proprietary file formats, but reconstructed volumes can be exported as image stacks in standard formats. The SkyScan 1174 images were reconstructed without binning and were stored as BMP image stacks. Both systems can employ a ring-artifact-reduction utility during reconstruction, and this was engaged for all the images presented here.

No single optimal set of parameters was used. Each type of sample is different, and, as with any kind of imaging, the requirements of the investigation at hand determine nature of the most useful images of a given sample. For the present applications it is useful to note that when using contrast agents with high x-ray absorption, the imaging is relatively insensitive to the anode voltage of the x-ray source. A higher voltage typically produces greater x-ray flux, which reduces the required projection exposure times and thus results in shorter scanning times. Details for each of the illustrated scans are given in Table 1.

**Table 1:** Details for the microCT scans illustrated in Figures. 2–15

**Fig.**	**Object**	**Fixation, Storage**	**Stain**	**Scanner**	**Voltage, Power**	**Mean Energy**	**Exp. Time**	**Scan Time**	**Voxel Size**
2	*Polyodon *head	Bouin's, 70% ethanol	PTA	Xradia	60 kV, 8 W	34 keV	11 sec	2.2 hrs	5.6 μm

3	*Polyodon *sections	Bouin's, 70% ethanol	PTA	Xradia	80 kV, 8 W	41 keV	14 sec	2.8 hrs	4.3 μm

3	grayling section	formalin	PTA	Xradia	40 kV, 8 W	26 keV	60 sec	12 hrs	2.1 μm

4	axolotl	glyoxal, 70% ethanol	PTA	Xradia	60 kV, 8 W	34 keV	15 sec	3 hrs	9.6 μm

5	pike hatchling	formalin	IKI	Xradia	30 kV, 6 W	22 keV	20 sec	4 hrs	4.0 μm

6	lamprey	formalin, 70% ethanol	I2E	SkyScan 1174	50 kV, 8 W	30 keV	1.6 sec 12	4.2 hrs	15 μm

7	*Polyodon *head	formalin, methanol	I2M	Xradia	80 kV, 8 W	41 keV	13 sec	2.6 hrs	5.1 μm

7	sturgeon pectoral fin	Dent's, methanol	I2M	Xradia	40 kV, 8 W	26 keV	10 sec	2 hrs	9.2 μm

8	*Xenopus *embryos	formalin	PTA IKI	Xradia	80 kV, 8 W 60 kV, 8 W	41 keV 34 keV	20 sec 25 sec	4 hrs 5 hrs	2.1 μm 3.2 μm

9	mouse embryo	paraform-aldehyde	IKI	Xradia	80 kV, 8 W	41 keV	13 sec	2.6 hrs	9.0 μm

9	mouse embryo	paraform-aldehyde	PTA	Xradia	60 kV, 4 W	34 keV	15 sec	3 hrs	9.6 μm

9	mouse embryo	EM fix, resin block	OsO_4_	Mayo Clinic	n.a.	n.a.	n.a.	n.a.	8.2 μm

10	insect thorax	Bouin's, 70% ethanol	I2E	Xradia	60 kV, 5 W	34 keV	20 sec	4 hrs	4.3 μm

10	insect head	Bouin's, 70% ethanol	I2E	Xradia	60 kV, 5 W	34 keV	80 sec	16 hrs	2.0 μm

11	insect tibia	Bouin's/alcohol, 70% ethanol	PTA	Xradia	60 kV, 4 W	34 keV	40 sec	8 hrs	0.9 μm

12	fly pupa	hot ethanol, 70% ethanol	PTA	SkyScan 1174	50 kV, 8 W	30 keV	1.6 sec 8	2.75 hrs	7.7 μm

13	*Falcidens*	EM fix, resin block	OsO_4_	Xradia	60 kV, 8W 80 kV, 8W	34 keV	5 sec 35 sec	1 hr 7 hrs	3.2 μm 1.6 μm

14	bryozoan *Cristatella*	Bouin's	PTA	Xradia	40 kV, 8 W	26 keV	5 sec	1 hr	4.2 μm

15	squid hatchlings	gluteraldehyde, cacodylate buffer	PTA IKI	Xradia	90 kV, 4W 40 kV, 6W	44 keV 26 keV	15 sec 20 sec	3 hrs 4 hrs	4.0 μm 4.4 μm

Both scanners used tungsten x-ray sources, and all scans were made over 180° rotation with images taken every 0.25° (thus reconstructions are based on 720 projections). The Xradia scanner can make single exposures from less than one second to several minutes, whereas the SkyScan 1174 uses a set exposure time of 1.6 seconds and averages multiple frames. The mean x-ray output energy for each anode voltage setting was estimated using the Siemens online tool for simulation of X-ray spectra . n.a. = data not available.

### Contrast stains and sample preparation

The most broadly useful contrast stains tested so far are inorganic iodine and phosphotungstic acid (PTA)[22]. The formulations and general procedures used are given in Table 2, and notes on the fixatives used are in Table 3[23-25]. The stains and procedures are simple and the procedures are robust. The staining times were found not to be critical, as long as the stain had sufficient time to penetrate the tissues. Inorganic iodine in alcoholic or aqueous solution diffuses rapidly into fixed tissues and was able to stain most specimens in a few hours or less, although staining was generally done overnight. PTA is a much larger molecule [26], and the solution used here was found to require overnight incubation to penetrate specimens 2–3 mm thick, and longer for larger specimens. PTA is known to bind heavily to various proteins and connective tissue [27,28], and this property, along with electron-shell energies that match common x-ray source emissions, suggested that it might be a useful stain for x-ray imaging. A few samples were tested with phosphomolybdic acid (PMA) staining, used similarly to PTA. The results (not shown) were generally similar, and PMA was not pursued further here (but see refs. [29] and [30] for successful application of PMA).

**Table 2:** Contrast stain formulations and protocols

**Stain**	**Stock solution**	**Staining procedure**
PTA	1% (w/v) phosphotungstic acid in water	Mix 30 ml 1% PTA solution + 70 ml absolute ethanol to make 0.3% PTA in 70% ethanol. Keeps indefinitely.
		Take samples to 70% ethanol.
		Stain overnight or longer.
		Change to 70% ethanol. Staining is stable for months.
		Scan samples in 70% – 100% ethanol

IKI	1% iodine metal (I_2_) + 2% potassium iodide (KI) in water	Dilute to 10% in water just before use.
		Rinse samples in water.
		Stain overnight.
		Wash in water.
		Can be scanned in water or dehydrated to alcohol.

I2E, I2M	1% iodine metal (I_2_) dissolved in 100% ethanol (I2E) or methanol (I2M)	Use at full concentration or dilute in absolute alcohol.
		Take samples to 100% alcohol.
		Stain overnight or longer.
		Wash in alcohol.
		Stain does not need to be completely washed out before scanning.

Osmium tetroxide	standard EM post-fixation	Same as routine EM processing.
		Osmium-stained samples can be scanned in resin blocks, with some loss of contrast.

Apart from osmium tetroxide, the reagents listed here are widely available, inexpensive, low-toxicity, and have been found to give good x-ray contrast to a wide variety of tissues. The stock solutions can be kept for months or longer at room temperature.

**Table 3:** Notes on fixatives

Fixative	Notes
neutral-buffered formalin (10% NBF)	Formalin = 37% formaldehyde solution (aq.).
	Normally used at 10% dilution in phosphate buffer at pH 7.0
	Commercial formalin usually contains about 10% methanol.
	The most common, but rarely the best fixative. [23,24]

paraformaldehyde	Polymerized formaldehyde, usually dissolved in buffer (e.g. PBS) at 4% w/v when a chemically-controlled fixative is required.
	Action is generally similar to 10% NBF. [23,24]

gluteraldehyde	Strong cross-linking fixative, often prepared in cacodylate buffer or a less toxic alternative such as HEPES. Common fixative for electron microscopy. [23,24]

4F1G	4% (or 3.7%) formaldehyde + 1% gluteraldehyde in phosphate buffer.
	Takes advantage of the faster penetration of formaldehyde and the superior fixing action of gluteraldehyde. Common fixation for electron microscopy. [25]

Bouin's fluid	75 parts (v/v) saturated aqueous picric acid,
	25 parts formalin (37% formaldehyde),
	5 parts glacial acetic acid.
	A standard and excellent histological fixative. [24]

alcoholic Bouin's	Refers to either a mixture of Bouin's fluid and ethanol (1:1), or to the fixative also known as Bouin-Duboscq-Brasil [24]. The two are similar in final composition.
	The alcoholic solutions penetrate more readily and are sometimes favored for arthropods.

glyoxal	A cross-linking dialdehyde (OCHCHO) prepared in acidic buffers and marketed as formalin substitutes: Prefer (Anatech Ltd.; ) and Shandon Glyo-Fixx (Thermo Scientific; ).
	Much less volatile and toxic than formaldehyde.
	Very good tissue preservation; especially good for immunostaining.

Dent's fixative	80% methanol, 20% DMSO
	Rapid dehydrating fixative. Expect some tissue shrinkage. Often used for immunostaining.

hot alcohol	Samples are dropped into 70% ethanol at about 60°C.
	Mainly used for fixing soft-bodied animals, such as insect larvae and pupae.

In general, the best fixative for microCT scanning will be the best histological fixative for the particular tissues under investigation.

### Sample mounting

**Figure 1:**
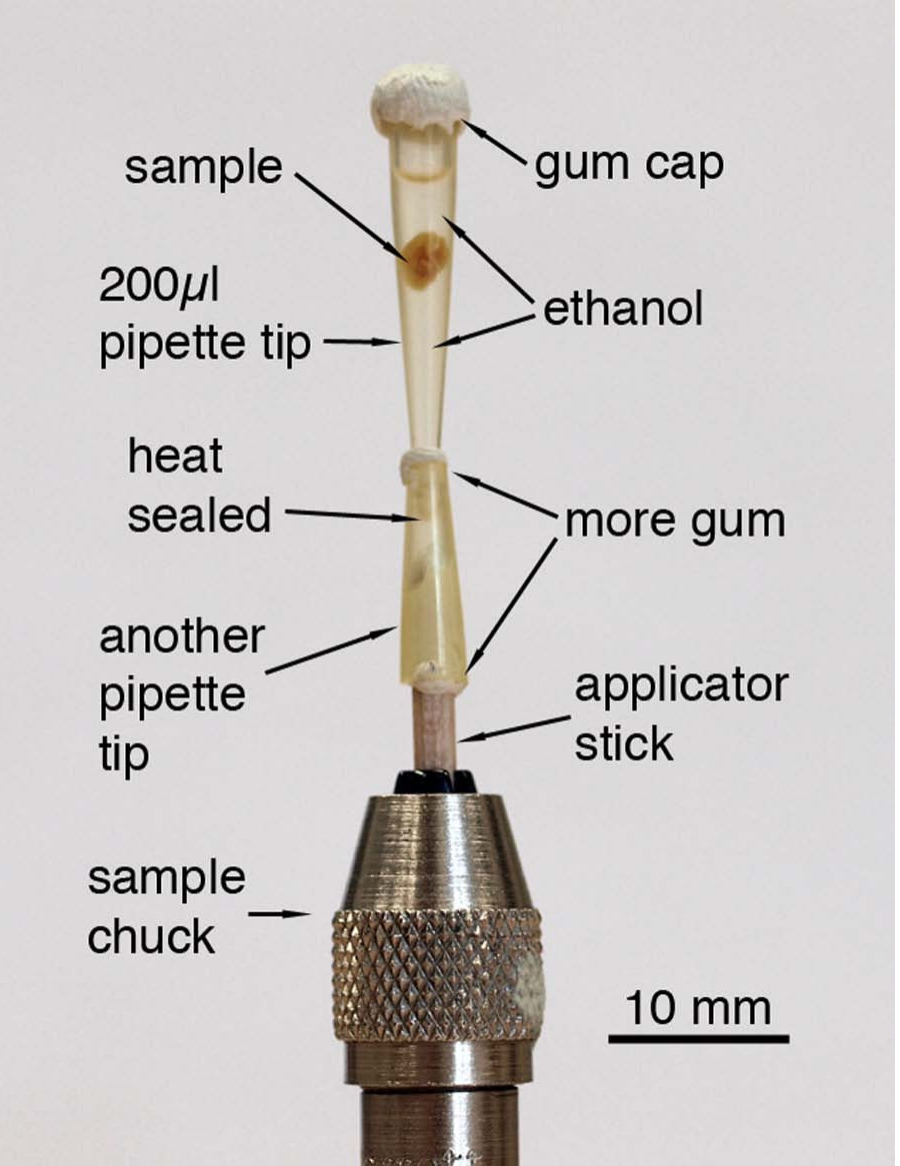
**Mounting arrangement for scanning samples in liquid**. A pipette tip is heat-sealed at its tip and filled with alcohol, and the sample is wedged gently into the tapering container. Adhesive putty such as UHU Patafix is used to prevent alcohol evaporation and also to hold the container tip in place in another cut-off tip mounted on a 2 mm-diameter wooden applicator stick, which is then mounted into the chuck supplied with the MicroXCT scanner.

All tissue samples that were not embedded in resin blocks were scanned in liquid media, most in absolute ethanol. To accomplish this, specimens were placed in small polypropylene tubes: either 0.2 ml PCR tubes, or heat-sealed pipette tips (Figure 1). Polypropylene has comparatively low x-ray absorption [31], and tips and tubes have thin (200–300 μm) walls. The conical shape of the container allows the sample to rest stably with a minimum amount of medium surrounding it. Absolute alcohol gave better tissue contrast than water, and alcohols have the added advantage of holding fewer bubbles due to lower surface tension. Bubbles in the tube with the specimen can expand and move during the scan, causing irreparable blurring in the reconstruction.

### Preparation of illustrations

The 3D viewing software provided with the Xradia scanner was used to produce the volume renderings and virtual sections for the illustrations. The window and level settings were adjusted for each image, and single frames were saved as PNG files. For volume renderings, the transparency function was also adjusted to show both internal and external features as appropriate. Images were cropped and arranged with Photoshop CS3, and further window and level adjustments were made using the Curves utility, and false color was added to the volume renderings. No further filtering or gamma corrections were performed on images of sections.

## Results and discussion

The results presented here reflect the author's experience with the samples and treatments tested so far, and these examples are intended to illustrate some possibilities for microCT investigations of diverse problems that require or will benefit from 3D morphological data. Among the most important practical results of this investigation is that each new type of sample must be tested with different fixations and stains to find the best treatment for the imaging required.

### Vertebrates

**Figure 2:**
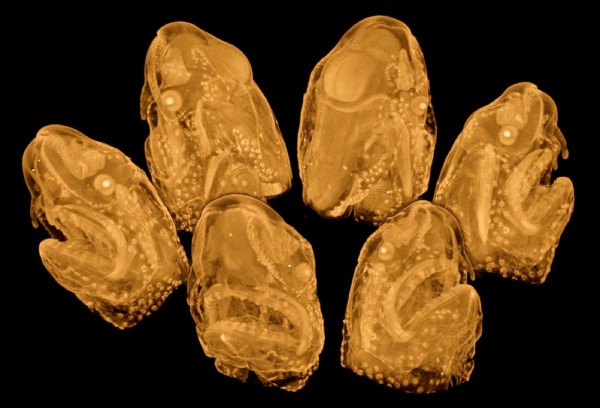
**Multiple views from a single scan of a 7-day post-hatching paddlefish (*Polyodon spathula*)**. Fixed in Bouin's, stored in 70% ethanol, stained with PTA. By adjusting the window and transparency functions, a volume rendering can be made to show a mix of internal and external structures. The lateral line receptors are especially prominent in these images, as are the nasal capsules and muscles. Voxel size 5.6 μm. Additional Files 1 and 2 are QuickTime movies of other PTA-stained paddlefish scans, one earlier (4 days post-hatching) and one later (27 mm total length).

**Figure 3:**
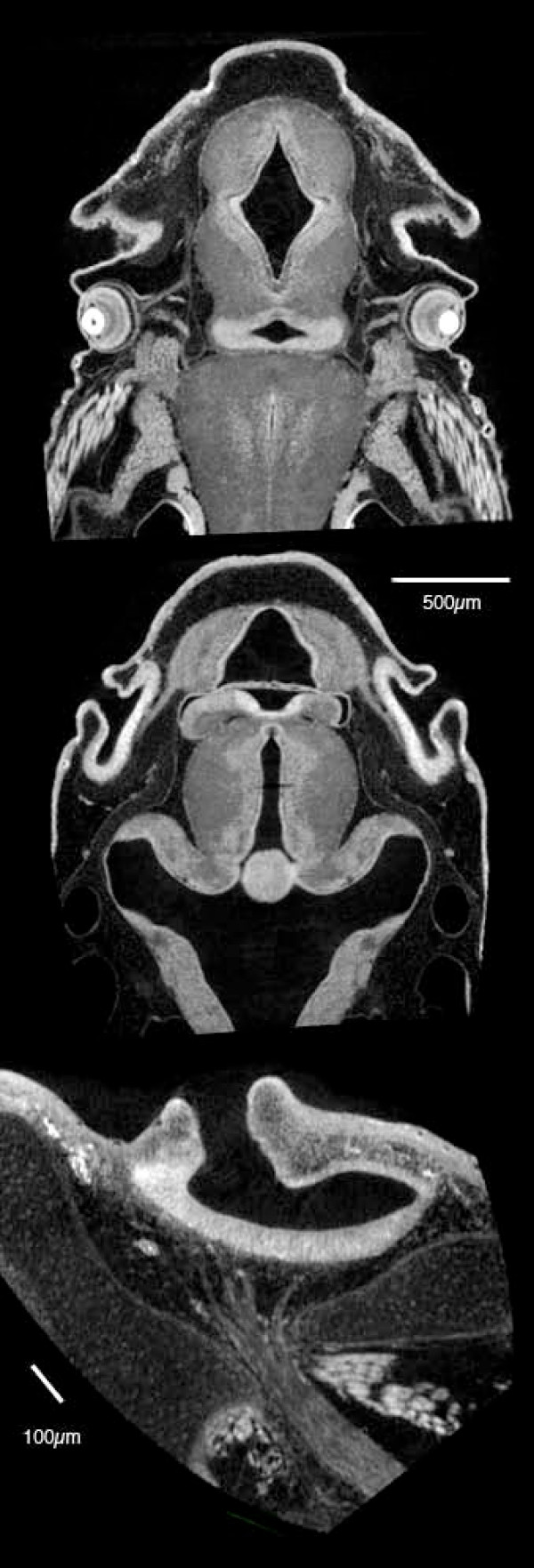
**Virtual sections of Bouin's-fixed, PTA-stained fish specimens**. Top two sections: *Polyodon*, 10 days post-hatching, horizontal sections through the head. Upper section shows the optic nerves, layers in the retina, and sections through the jaw adductor muscles. Middle section is more dorsal and shows neurocranial cartilage and otic chambers. Both sections show differential staining of brain tissues and the olfactory organs. Voxel size 4.3 μm. Bottom: European grayling (*Thymallus thymallus*) fixed in formalin and stained with PTA. This section shows the external naris (at top), the olfactory epithelium, and the olfactory nerve, as well as cranial cartilage. Voxel size 2.1 μm.

**Figure 4:**
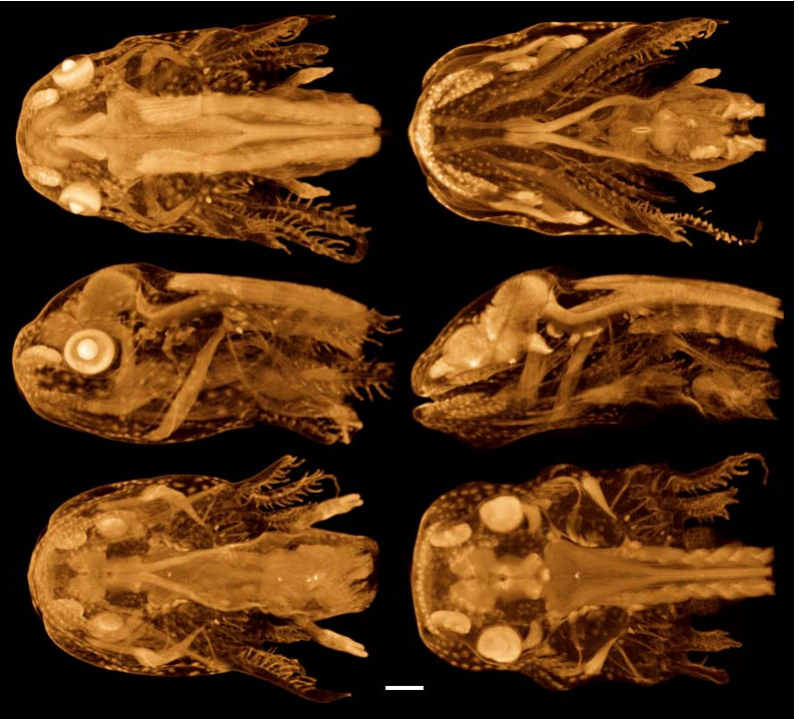
**3D renderings of an axolotl (*Ambystoma mexicanum*) feeding larva**. Glyoxal-fixed, stored in 70% ethanol, PTA-stained. Left column shows external views from the dorsal, left, and ventral sides, each with the background half deleted for clarity. Right column shows the same views, but with the foreground half removed. PTA staining highlights muscles and nervous tissues, as well as sensory organs: note the prominent nasal capsules and neuromasts. Horizontal bar = 500 μm. Voxel size 9.6 μm.

**Figure 5:**
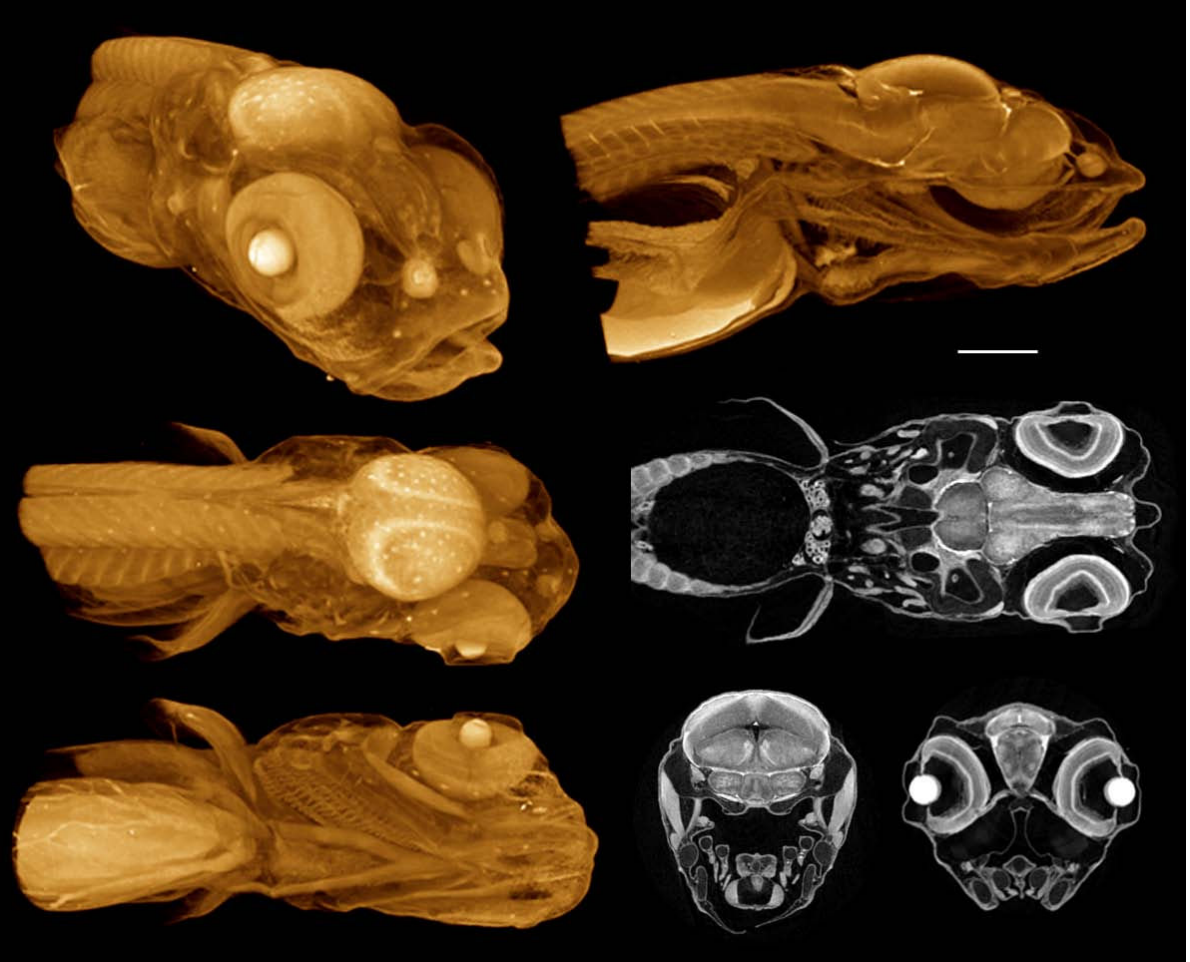
**Pike *(Esox lucius)* fry fixed in formalin and stained with IKI**.  Volume renderings and virtual sections made from the concatenated stacks of  reconstructed slices from two microCT scans made with the sample on the same rotation  axis (i.e. translated only in the anterior-posterior direction), one scan of the fish’s head  and the other of the pectoral region. This procedure allowed higher-resolution scanning of  both body regions (at the expense of extra scanning time). Left: External views of an  overall volume rendering, with the transparency adjusted to reveal some internal  structures. Top right: Midsagittal cutaway of the same volume rendering. Center right:  Single-voxel horizontal virtual section through the volume image. Bottom right: Coronal  sections through the same volume image stack. One shows layers in the brain, sections  through the jaw adductor muscles, and gill-arch cartilages; the other shows retinal layers,  connections with the optic nerves, and the lenses, which tend to stain very heavily.  Horizontal bar = 500 μm. Voxel size 4.0 μm. Additional File 3 is a QuickTime movie of  the a volume reconstruction from these scans.

The PTA and iodine stains were found to impart strong tissue contrast to fish and amphibian samples. Especially good results were obtained with PTA staining of Bouin's- or glyoxal-fixed material (Figures 2, 3 and 4), and with IKI staining after formalin-fixation (Figure 5). PTA is known to bind to collagen and other proteins [28], and musculature is demonstrated distinctly in tomographic images, along with other structures (Figures 2, 4, Additional files 1, 2). Cartilage does not stain strongly with PTA, but appears as gaps in volume renderings. However, the individual hypertrophic chondrocytes within the cartilage matrix can be seen clearly in high-resolution virtual sections (Figure 3, bottom). IKI (10% aq.) applied to samples still in aqueous medium imparted differential x-ray contrast at least as high as that obtain with PTA. Nervous tissues are also demonstrated well with both stains, and different layers of the brain can be distinguished easily (Figures 3, 5, Additional file 3).

**Pike (*Esox lucius*) fry fixed in formalin and stained with IKI**. Volume renderings and virtual sections made from the concatenated stacks of reconstructed slices from two microCT scans made with the sample on the same rotation axis (i.e. translated only in the anterior-posterior direction), one scan of the fish's head and the other of the pectoral region. This procedure allowed higher-resolution scanning of both body regions (at the expense of extra scanning time). Left: External views of an overall volume rendering, with the transparency adjusted to reveal some internal structures. Top right: Midsagittal cutaway of the same volume rendering. Center right: Single-voxel horizontal virtual section through the volume image. Bottom right: Coronal sections through the same volume image stack. One shows layers in the brain, sections through the jaw adductor muscles, and gill-arch cartilages; the other shows retinal layers, connections with the optic nerves, and the lenses, which tend to stain very heavily. Horizontal bar = 500 μm. Voxel size 4.0 μm. Additional File 3 is a QuickTime movie of the a volume reconstruction from these scans.

**Figure 6:**
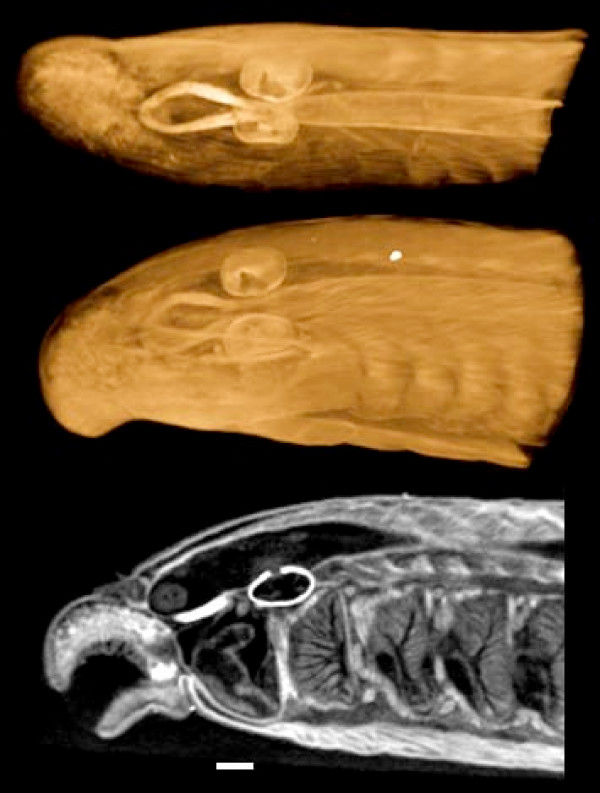
**Juvenile lamprey (*Lampetra*), scanned with the SkyScan 1174**. Fixed in formalin and stained with I2E after storage in alcohol. Whole specimen was approximately 10 cm in length. Top: ventral view of a horizontal cutaway volume rendering. Center: external view of the head region. Bottom: single reconstructed parasagittal section. Voxel size 15 μm. Sample provided by Daniel Sidertis, Univ. Vienna.

**Figure 7:**
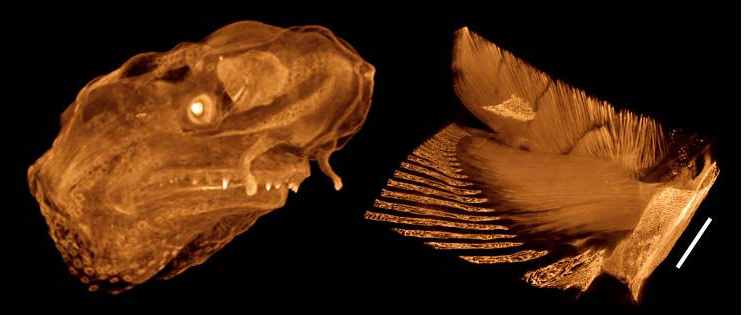
**Iodine (I2M) staining of methanol-preserved samples**. Left: Paddlefish *Polyodon spathula*, 11 days post-hatching, formalin-fixed and methanol dehydrated for in situ hybridization, stained with I2M. Voxel size 5.1 μm. Right: Green sturgeon (*Acipenser medirostris*, 65 mm total length; kindly provided by Boyd Kynard of the Conte Anadromous Fish Laboratory, MA) fixed in Dent's and stored in methanol, stained with I2M to demonstrate the pectoral musculature. Iodine tends to overstain calcified tissues: x-ray absorption by bone, scutes, and lepidotrichia is outside the image brightness window in these images. Scale bar = 1 mm. Voxel size 9.2 μm.

For samples stored in alcohol, effective staining was obtained with 1% iodine metal in absolute ethanol or methanol (I2E or I2M; see Table 2). This method is especially useful for archival or museum specimens stored in 70–95% ethanol (e.g. the lamprey in Figure 6), and for samples preserved in methanol as for in situ hybridization (e.g. the paddlefish in Figure 7). The effects of previous dehydration are evident, and the staining is slightly less distinct than with treatments on samples fixed for the purpose of microCT imaging. It is worth noting that iodine did not stain effectively in 70% alcohol, and so samples had to be transferred to 100% alcohol before staining.

Accurately calibrated 3D images of musculoskeletal systems can be also used to quantify muscle fiber numbers and cross sectional areas, muscle attachment areas, bone or cartilage sizes and shapes, and facilitate functional modeling (as in [32]).

### Vertebrate Embryos

**Figure 8:**
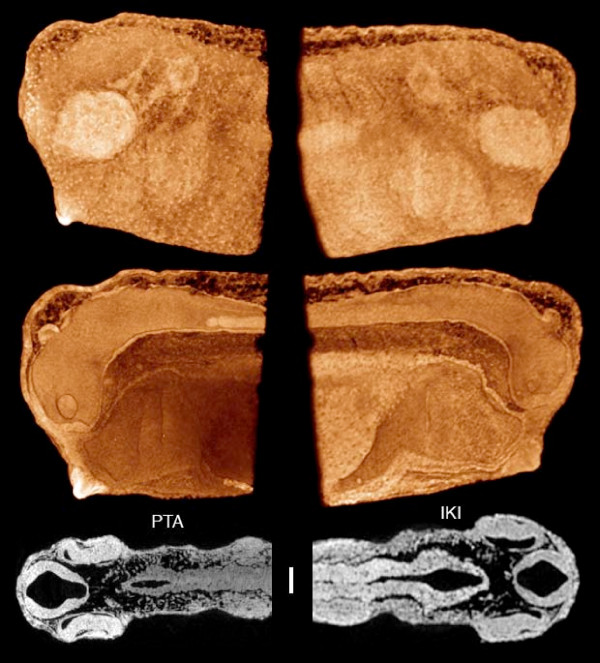
***Xenopus *embryos, stage ca. 27**. Fixed in formalin and stained with PTA (left) and IKI (right). Bottom: virtual 1-voxel-thick horizontal sections; scale bar = 100 μm. Voxel size 2.1 μm (left) and 3.2 μm (right). Samples provided by Daniel Sidertis, Univ. Vienna.

A separate report has already described and evaluated the methods employed here for staining and imaging chick embryos with microCT [22]. For the present report a few formalin-fixed *Xenopus *embryos were stained with IKI and with PTA, with fairly similar results (Figure 8). General feature such as pharyngeal pouches, sucker, and otic and optic vesicles are clearly distinguishable, as are ciliated epidermal cells, the epiphysis, and differentiating neural tube.

**Figure 9:**
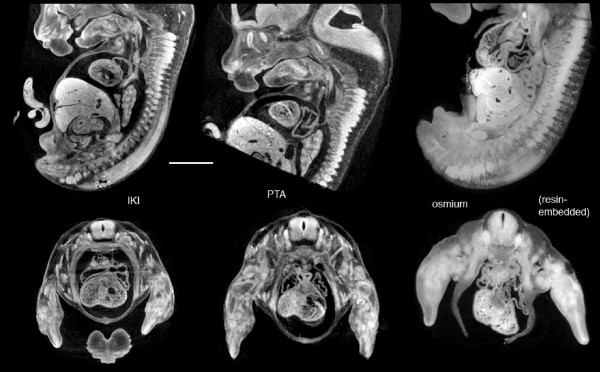
**Mouse embryos**. Left: Virtual sections of a Theiler stage 21 mouse, paraformaldehyde-fixed and IKI-stained. Voxel size 9.0 μm. Center: Similar embryo, PTA-stained. Voxel size 9.6 μm. Right: Osmium-stained 12.5 dpc mouse embryo embedded and scanned in JB4 resin (standard TEM preparation). Voxel size 8.2 μm. Sample was prepared by R. Walsh (Life Sciences EM Facility, Penn State University), and the microCT scan was made in 2001 at the Mayo Clinic in the laboratory of Dr. Erik Ritman (Physiological Imaging Research Lab, Rochester MN). Additional Files 4 and 5 are QuickTime movies of this PTA-stained embryo and a stage 22 IKI-stained embryo.

Paraformaldehyde-fixed mouse embryos gave strong overall contrast with both IKI and PTA staining (Figure 9 and Additional files 4, 5, 6). The general differentiation among tissues is at least comparable to that obtained with critical-point drying [33] or osmium staining [15], though perhaps not as fine as in some microMRI images [34].

Osmium stained tissues can be imaged with microCT after embedding in resin, as for TEM sectioning. Figure 9 (right) shows virtual sections from an osmium stained 12.5 day mouse embryo in JB4, and scanned in 2001 on the in-house scanner at the Mayo Clinic (Rochester, MN). The differences in absorbance levels between different organs are not quite as high as those in the IKI and PTA samples, likely due in part to the infiltrated resin. This opens the possibility of microCT imaging and TEM with the same embedded sample, providing exact registration between ultrastructure and quantitative whole-volume images.

To date, osmium tetroxide has been the most common contrast stain for microCT imaging of soft tissues, and it is a natural candidate: osmium has electron binding energies favorable for strong x-ray absorption, it is already available in many institutions, and osmium is known to bind to cell membranes and other lipid-rich structures including nerves. However, osmium is very toxic, expensive to dispose of, and does not stain well if samples have been in alcohol. Its penetration is slow, and may reach an upper limit in specimens larger than a mid-gestation mouse [15,27]. PTA penetrates tissues slowly also, but it is far less toxic, much simpler to use, and will effectively stain alcohol-stored samples. Inorganic iodine readily penetrates all soft tissues tested so far, and it has proven to be versatile and robust contrast stain.

### Insects

MicroCT is already becoming established as a method for imaging insect morphology [35]. Contrast methods currently in use include phase contrast [11], staining with osmium and other heavy metals [16,17,36], and critical-point drying [37,38]. The methods presented here offer additional options, especially when it is desirable to scan samples in liquid or to emphasize different subsets of tissues.

**Figure 10:**
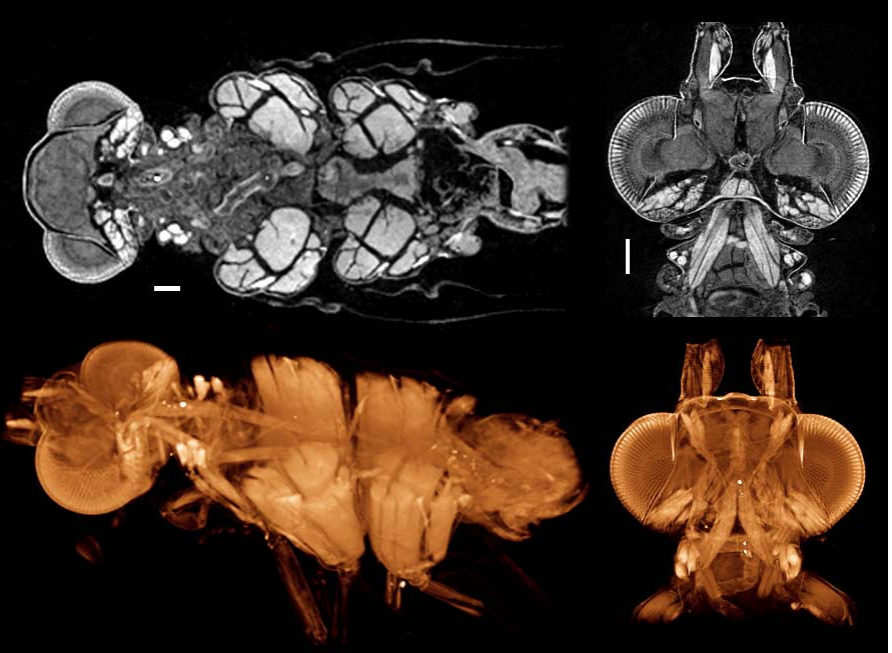
**A neuropteran insect (genus *Sisyra*) showing musculature and general soft tissue morphology**. Fixed in Bouin's fluid and stained with I2E, scanned in ethanol. Virtual horizontal sections one voxel thick (top), and cutaway volume renderings (bottom). Muscles are especially clearly defined, and the complete penetration of iodine makes it more suitable than PTA or osmium for whole insect specimens. Scale bars, 100 μm. Samples from Dominique Zimmermann, Natural History Museum, Vienna. Voxel size 4.3 μm (head + thorax, left), 2.0 μm (head, right).

Iodine-stained insects scanned in alcohol show detailed structure of both chitinous and soft tissues while preserving their native spatial arrangements (Figure 10). Musculature is especially clear in images of I2E-stained insects after Bouin's fixation: the origin, course, and insertion of each muscle is precisely represented, and such images will allow quantitative measurements of muscle cross sections and attachment areas, as well as offering the possibility of functional modeling. I2E staining also worked well on insects fixed only in alcohol, although the overall preservation of morphology is superior with Bouin's fixation.

**Figure 11:**
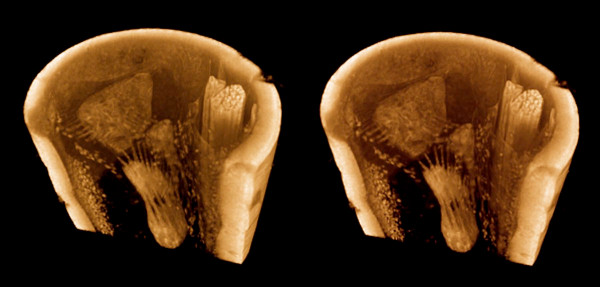
**Tibia of a mantophasmid insect (undescribed genus), showing the vibration-sensitive scolopidial organ**. Stereo pair for convergent (cross-eyed) viewing. Bouin's/alcohol fixed, PTA-stained. It was necessary to cut segments of leg no longer than 3–4 mm to allow the PTA to penetrate through. Diameter of the leg is approx. 300 μm. This volume rendering shows the scolopidial organ, and individual sensory cells and fibers, muscle fibers, and single blood cells can be resolved clearly. Insect chitin stains more heavily with PTA than with iodine. Sample from Monika Eberhard, Univ. Vienna. Voxel size 0.9 μm.

In an investigation of sensory organs in mantophasmid legs (ongoing collaboration with M. Eberhard, Univ. Vienna), PTA staining after fixation in alcoholic Bouin's was found to give clearer differentiation of fine-scale structures than iodine in microCT images (Figure 11). The PTA did not readily penetrate the cuticle, so insect legs had to be cut to lengths of no more a few millimeters to allow the stain to penetrate into the tissues of interest. Individual sensory cells and fibers can be seen within the scolopidial organ, as well as single blood cells and individual muscle fibers.

### Insect pupae

Alcoholic iodine and PTA were tested on fly pupae of different stages as part of an ongoing study of flesh fly metamorphosis (with M. Forcher, Univ. Vienna). Fixation in hot ethanol was found to work well for preserving pupal morphology for staining and imaging. Adequate staining was obtained only by puncturing or even removing the puparium to allow the stain solution to penetrate, especially for PTA staining.

**Figure 12:**
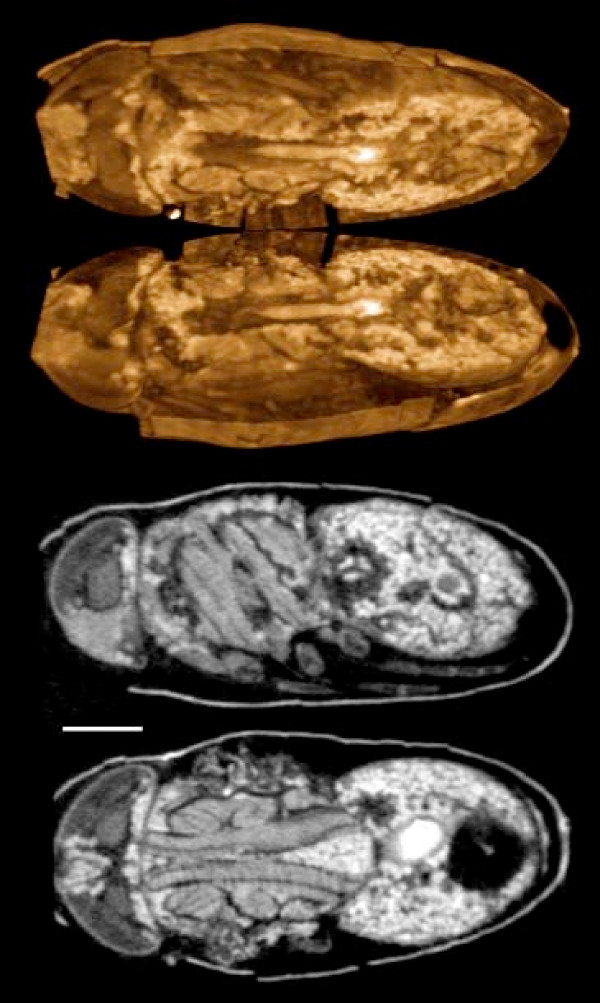
**Late pupa of the flesh fly *Calliphora vicinia *(Diptera)**. Fixed in hot ethanol and stained with PTA. Pupae must be perforated for PTA to penetrate. Top: volume rendering with a horizontal cutaway. Bottom: parasagittal and horizontal single reconstructed sections. Scale bar: 1 mm. Samples prepared and provided by Marlis Forcher, Univ. Vienna. SkyScan 1174 scan, voxel size 7.7 μm.

Figure 12 shows a late-stage pupa (one day before eclosion) of the flesh fly *Calliphora *(Diptera: Calliphoridae) stained with PTA. The staining is complete, and the insect's near-adult morphology is clearly visible. Due to their size – about 8 mm long – these pupae were imaged using the SkyScan 1174.

### Other invertebrates

**Figure 13:**
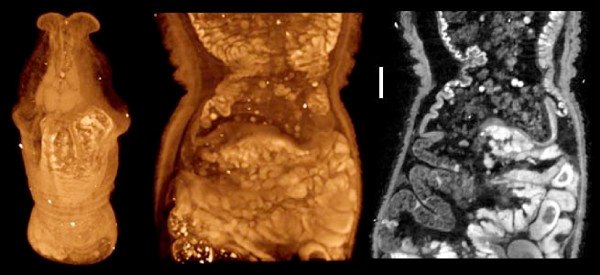
**A caudofoveate mollusc (*Falcidens sp*)**. Stained with osmium tetroxide and embedded in Spurr's resin for electron microscopy, scanned in resin block. Volume rendering (left) of a low-resolution scan, showing the anterior-most 1.4 mm of the animal. Cutaway volume rendering (center) and a single reconstructed section (right) of a higher resolution scan of the same block, showing the midgut region. This volume image was processed with a median filter (3 × 3 kernel). Scale bar = 100 μm. Voxel size 3.2 μm (left), 1.6 μm (center and right). Sample prepared and provided by Emanuel Redl, Univ. Vienna.

**Figure 14:**
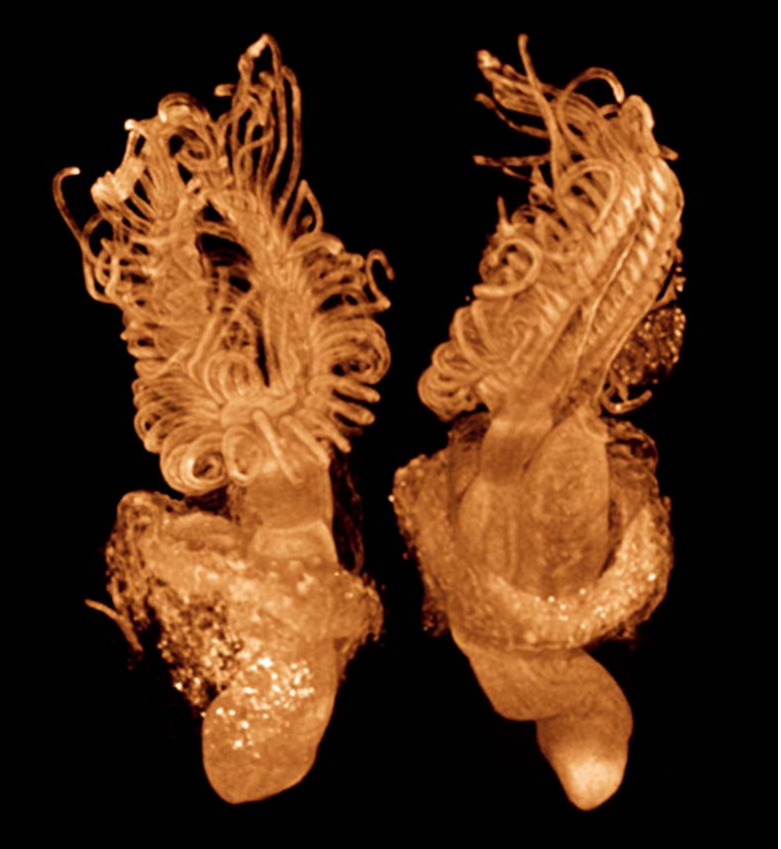
**Volume rendering of the freshwater bryozoan *Cristatella mucedo *stained with PTA**. Specimen was fixed in Bouin's and stained with PTA. Scanned in alcohol. Total length is approximately 2 mm. Voxel size 4.2 μm. Sample from Thomas Schwaha and Stephan Handschuh, Univ. Vienna.

**Figure 15:**
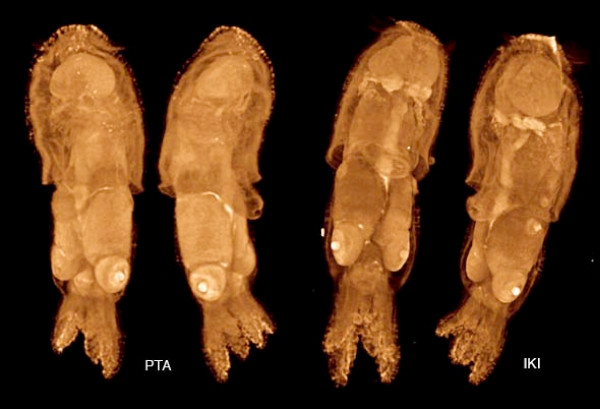
**Squid hatchlings, *Ideosepius pygmeus*, ca. 2 mm long**. Fixed in gluteraldehyde, stored in cacodylate buffer, and stained with PTA (left) and IKI (right). The IKI staining required less than one hour. Voxel size 4.0 μm (left) and 4.4 μm (right). Samples from Janek von Byern, Univ. Vienna.

The methods presented here are not species-specific, and a few other examples are shown in Figures 13, 14 and 15. A caudofoveate mollusk of the genus *Falcidens *was stained with osmium and embedded in Spurr's resin, a standard method for sectioning and TEM imaging. Figure 13 shows images from a microCT scan of the intact resin block. Because the volume image can be virtually re-sectioned in any arbitrary plane, subsequent TEM images can be matched to their original locations in the whole specimen and accurately correlated with the overall picture of the animal.

In Figure 14, volume renderings of a freshwater bryozoan (*Cristatella mucedo*) show the spatial relationships among all the structures in the specimen, with the potential for quantitative measurements and extraction of soft-tissue characters important for systematics [29].

The squid hatchlings in Figure 15 were stained with PTA (left) and IKI (right) and scanned in alcohol. The two stains give somewhat different patterns of tissue differentiation in x-ray images, emphasizing the importance of testing different stains on each new kind of sample. Recently, Golding et al. [29,30] have used phosphomolybdic acid (PMA) staining to enhance contrast in gastropod mollusks for microCT scanning and segmentation of the odontophoral cartilages. Their results indicate that the use of PMA as a microCT stain may deserve further attention.

## Conclusion

Using a few simple contrast stains, microCT can provide versatile, high-contrast, quantitative 3D images of animal soft tissues. The methods can be used on a wide variety of animal specimens fixed and preserved by the most common methods. These stains are easy to use and low-toxicity, and obviate the need for critical-point drying. Stained tissues can be scanned in liquid, and further analyses can be performed on the same samples. Versatile x-ray contrast stains add to the options for volumetric imaging of non-mineralized tissues, and it is hoped that they will prove useful in comparative, developmental, and evolutionary research.

## Competing interests

The author declares that they have no competing interests.

## Supplementary Material

Additional file 1 **Polyodon spathula, 4 days post-hatching**.QuickTime movie of volume rendering. Bouin’s fixed, PTA stained. Click here for file

Additional file 2 **Polyodon 27mm total length**. QuickTime movie of volume rendering. Bouin’s fixed, PTA stained. Click here for file

Additional file 3 **Pike (Esox lucius) hatchling**. QuickTime movie of volume rendering from two concatenated scans. Formalin-fixed, IKI-stained. Click here for file

Additional file 4 **Mouse embryo, Theiler stage 21**.QuickTime movie of volume rendering. Paraformaldehyde-fixed, PTAstained. Click here for file

Additional file 5 **Mouse embryo, Theiler stage 22**. QuickTime movie of volume rendering of two scans concatenated to show the whole embryo. Paraformaldehyde-fixed, IKI-stained. Click here for file

Additional file 6 **Paraformaldehyde-fixed mouse embryos**. Click here for file
